# Pawsitive Impact: Measuring the Dog Mentor’s Effect in Neurodivergent Students

**DOI:** 10.3390/bs16030323

**Published:** 2026-02-26

**Authors:** Mirena Dimolareva, Ella Doolan-Dransfield, Jenny Duckworth, Victoria L. Brelsford, Kerstin Meints, Nancy R. Gee

**Affiliations:** 1 School of Psychology, Sport Science and Wellbeing, University of Lincoln, Lincoln LN5 7AY, UK; vbrelsford@lincoln.ac.uk (V.L.B.); kmeints@lincoln.ac.uk (K.M.); 2 Division of Psychology and Language Sciences, University College London, London WC1E 6BT, UK; ella.doolan-dransfield.23@ucl.ac.uk; 3 The Dog Mentor, Rochester ME3 8RU, UK; jen@thedogmentor.co.uk; 4 Center for Human-Animal Interaction (CHAI), Virginia Commonwealth University, Richmond, VA 23298, USA; nancy.gee@vcuhealth.org; 5 Department of Psychiatry, Virginia Commonwealth University, Richmond, VA 23298, USA

**Keywords:** autism, education, school, Animal-Assisted Services and Interventions, dogs

## Abstract

Children diagnosed with autism face many barriers to learning. Animal Assisted Services and Interventions (AAS/AAI) have been adopted to support children within schools. The Dog Mentor is a UK-based organisation that provides training for handlers and assesses dogs to be integrated within schools. It adopts a rigorous and continuous training package and ensures the safety and welfare of all involved by adopting a whole school approach. This research uses content analysis to understand the types of activities and outcomes in The Dog Mentor programme, as established by teachers and dog handlers, across 58 schools. Teachers and dog handlers perceived that The Dog Mentor successfully supported children with autism, using a variety of sessions. This variability is seen as a benefit as it enables the intervention to be tailored to meet the needs of the students. Handler-reported benefits include creating a calm environment, promoting engagement, and supporting learning. Improved self- and emotion regulation, mental health, and resilience were also noted by the handler reports. Future research needs to investigate these perceived benefits using quantitative data, as well as look into outcomes relating to the dogs supporting others with bereavement and trauma. This topic was briefly mentioned by two of the schools, but there was not enough data to understand the impact in depth.

## 1. Introduction

Neurodivergent people experience and interact with the world differently than neurotypical individuals, owing to how they process information, resulting in a difference with learning, behaviour, and daily tasks (National Autistic Society, 2025; Benderix et al., 2006). Neurodivergent conditions cover a wide spectrum of abilities and challenges that individuals face, including Autism Spectrum Disorder (ASD) (McGee, 2012). Within the UK, one in 100 people is on the autism spectrum, a lifelong neurodevelopmental condition, varying in severity and characteristics (National Autistic Society, 2025; CDC, 2024). For children diagnosed with autism, difficulties in communication and delays in development are witnessed, with recommended early interventions (Landa, 2007; Cheng et al., 2023). Yet delays in diagnosis due to long waiting lists are common within the UK (British Medical Association, 2024). Upon diagnosis, further delays are experienced when obtaining an Educational Health Care Plan (EHCP), which is essential for outlining the child’s needs in relation to sensory sensitivities, communicative difficulties, and challenging behaviours, creating significant barriers to learning (McDougal et al., 2020; Naznin et al., 2023). The deadline for completion of an EHCP is 20 weeks, but only half are issued within this timeframe (UK Parliament, 2024). These delays deny the opportunity for the recommended early intervention and support, essential for removing barriers to facilitate children’s learning (British Medical Association, 2024; Machelicek et al., 2007).

To facilitate learning, schools utilise a variety of support strategies and interventions, such as social stories to teach social skills and change the learning environment, aiming to engage the child and enhance learning (Gray & Garand, 1993; Machelicek et al., 2007). More recently, the use of Animal-Assisted Services and Interventions (AAS/AAI) has become more popular. Improvements in autism symptoms, social interaction, social functioning, and communication in children on the autism spectrum have been demonstrated (Dimolareva & Dunn, 2021; Meints et al., 2022; Sissons et al., 2022; Gabriels et al., 2015, 2018). Within educational settings, school staff have positive perceptions of the implementation of AAIs to support mental health (Leos et al., 2022). AAS/AAIs have shown to improve psychological, social, and academic outcomes, creating a positive school environment, with dogs assisting students in regulating their emotions and behaviours, yet outcomes relating to improvement of social skills have been mixed (Zents et al., 2017; Baird et al., 2023; Wintermantel et al., 2024; O’Haire et al., 2014; Anderson & Meints, 2016). Furthermore, there is significant variability in AAS/AAI sessions, including the structure within the sessions and frequency per week and overall duration (Heimlich, 2001; Somervill et al., 2009; Limond et al., 1997; Martin & Farnum, 2002). Though variations can be beneficial, to address various needs, it makes generalisation and guidance around effectiveness difficult.

The Dog Mentor is a UK-based organisation that assesses school dogs and provides training for handlers and wider academic support to integrate animals within the curriculum delivery. Over 600 schools are registered with the organisation, which all employ a whole school approach to ensure the well-being of the dog is not compromised. This includes the use of strategies to regulate children’s behaviour and a set way to behave throughout the school, regardless of whether the dog is currently there. For instance, at lunch, children take extra care not to drop food, as this can make the dog sick if they consume it. Another example is the children are taught “Dog Mentor breathing”, which gets them to think about their breathing, de-escalating a behaviour, and helping them to physically regulate in a challenging situation. The training for all staff working with the dog mentors is based on positive reward, empathy, respect for the animal, and children’s self-regulation prior to interaction. As part of the registration, a rigorous risk assessment is completed, specific to the educational environment and dog(s). The registered schools also have access to dog-related materials and activities they can integrate into their curriculum. Meanwhile, there is flexibility for school staff to incorporate the work with the dogs as necessary providing the overall risk assessment is followed. This is a key element of The Dog Mentor to ensure the safety of all and optimal outcomes. As the programme has grown, schools have shared many case studies of impact that the dogs have had. The variability seen in the research field is also evident within The Dog Mentor, but here, we have a large sample of schools that follow the same basic principles. As a result, we are able to develop research and look at different aspects of the AAS/AAI and their effectiveness. For a first qualitative evaluation of the impact of the programme, the current paper presents questionnaire data from the schools that have incorporated work with the dogs for their children on the autism spectrum. Content analysis has been used to answer the following questions:

1. To what extent is The Dog Mentor effective at reducing the symptoms experienced by children on the autism spectrum?
2. How can The Dog Mentor be integrated to effectively reduce the symptoms experienced by children on the autism spectrum?
3. To what extent is The Dog Mentor effective at facilitating learning for children on the autism spectrum?

## 2. Materials and Methods

### 2.1. Data Collection

The Dog Mentor team regularly contacts the schools within the programme to keep updated on the work that is ongoing and to provide support with any challenges and additional materials for lessons. One questionnaire the schools received related to the work and challenges of incorporating the dog into the educational process with diagnosed and undiagnosed neurodivergent children. The data was anonymised by JD (founder of The Dog Mentor) and sent to the research team for analysis.

The Dog Mentor aims to support schools in incorporating dogs within their settings. As a result, it is driven by the needs of the school, and there is no typical length or duration of each setting. Some schools have had their dog only when a specific need arises- for example, a bereavement in the family resulting in a child struggling to go to school, so the dog has been included in the routine to support the child in going to school. On the opposite end of the scale, some dogs are in school daily (and some schools have more than one dog) and have a timetable with different activities and rest periods. Here, it is also vital to note that the activity of the dog depends on the age, personality, and energy of the dog, too. This is part of the initial assessment and is reviewed regularly to ensure dogs are not overworked. In addition to that, The Dog Mentor is adaptive in nature, so more guidance is added when challenges occur. For instance, the safety of the dog is paramount, so there are clear instructions on how to lead a dog down a corridor and through fire (and other) doors, to ensure the dog is not either stroked by children/crowded or trapped/hurt by a door.

### 2.2. Participants

Fifty-eight schools responded to the questionnaire, including primary schools (n = 30), Special Educational Needs schools (SEN)/Specialist Provision (n = 12), secondary schools (n = 9), through schools which include nursery to 18 years (n = 3), primary schools with nursery schools (n = 2), infant schools (n = 1) and a primary and secondary school (n = 1). No individual-level diagnostic, severity, or demographic data were collected for specific children. Respondents answered based on their experience with students whom they understood to be on the autism spectrum and/or otherwise neurodivergent, including both formally diagnosed and undiagnosed pupils. All responses were provided by the dog handlers, who are employed by the school, and being a handler is an additional role to their primary job (e.g., teaching assistant, teacher, SENCO). One respondent was from the finance department of the school, but the remaining respondents (n = 57) were all teaching staff. All respondents therefore had a good understanding of the outcomes they had seen in the children with a variety of interventions, including the Dog Mentor and other non-animal related interventions, for comparison. No financial incentive was provided, and the questionnaire was only sent once to avoid pressuring respondents.

### 2.3. Questionnaire

The questionnaire was developed by the Dog Mentor team, and the respondents did not have to answer all the questions. At the end of each question below, we have indicated how many of the handlers responded to each question. The questionnaire included the following questions:

- Have you been able to use your Dog Mentor(s) to achieve any of the following: when you have worked with children with autism or undiagnosed neurodiversity? The options were based on the training provided by The Dog Mentor and were: reducing stress and anxiety; promoting a more productive environment for learning; curriculum support; help with social interactions; build long term rapport; promote empathy and a caring attitude within school; teaching responsibility; teaching and creating positive body movements; supporting with communication; teaching self-care and life skills; develop and establish acceptance understand constructive criticism; self-awareness; self-regulation; empathy; focus and on task behaviour; resilience; dissolve attachment seeking behaviour; exploring ideas, environments, subjects; support with sensory issues; support with daily structure and routines; support with making mistakes and natural consequences; support with restoring relationships after challenging situations; promote positive authentic environments; promoting relaxed classrooms; improve engagement; positive environment (58/58 responses)
- Thinking specifically of the children with autism that your Dog Mentor has been involved with, what activities did the dog participate in? (57/58 responses)
- Thinking specifically of the children with autism that your Dog Mentor has been involved with, can you provide more details on the aims of the activities you completed? (52/58 responses)
- Do you think the dog has been useful in achieving the goals you set out to achieve when working with the children with autism? (55/58 responses) Please let us know why you chose the above answer. (49/58 responses)
- What would you say the biggest challenge was when incorporating the Dog Mentor within the tasks with children with autism? (53/58 responses)

The aim was to keep the questionnaire brief, with open-ended questions so all respondents could provide as much detail as they felt was necessary. This also ensured that even if teachers had little time, they could still provide some information on the work they were doing. It is also important to note that not only did respondents not have to answer all questions, but not all schools that are part of The Dog Mentor completed this questionnaire. This may have been due to them not including the dogs to work with neurodivergent children, or because they have not seen a benefit in doing so, and, therefore, felt they did not have anything to share. As the current questionnaire is framed to ask for best practice in order to find out ‘what works’, it is likely that we have more positive responses in the effectiveness of The Dog Mentor within the neurodivergent population. However, this allows us to explore this avenue and provide guidance to schools, as well as complete a larger-scale evaluation of the effectiveness of this work. This will be incorporated in later research with a larger, more scientifically rigorous design.

### 2.4. Analysis and Coding

Two researchers (EDD and JD) analysed the data via content analysis, following a four-step procedure. Raw data was read through to understand the contexts of the data before a hybrid coding approach was undertaken. Deductively, a tally was completed for results gained for question one to assess the frequency of responses, and inductively, new codes were developed from the raw data for the remaining questions. Codes were then grouped together through content relationship, creating sub-themes, and additionally tabulated for frequency. Finally, sub-themes were grouped together in similarity, creating overall common themes.

#### Intercoder Reliability

The data were independently coded by two of the authors trained in content analysis (EDD, JD), one with further training in qualitative methods (EDD–MRes in Social Research). Themes uncovered were compared and achieved a percent agreement of 90% inter-rater reliability. Trauma and loss support was selected as a possible theme by JD, but due to the infrequency of mentions (seven total occurrences across two schools), its exclusion was agreed upon by the coders in the final write-up. The team agreed that this theme should be explored in future research to understand the impact of AAS/AAI for children on the autism spectrum experiencing trauma and loss.

## 3. Results

Almost all the schools (93%) reported The Dog Mentors’ usefulness in achieving their aims in supporting children on the autism spectrum, utilising a variety of interventions (Table 1), and improving three key areas: learning, mental health, and relationships.

### 3.1. Theme 1: Facilitated Learning

All of the schools reported an improvement in learning with the dog mentors, creating a positive environment and supporting overall learning.

#### 3.1.1. Subtheme: Creating a Positive Environment

The most commonly reported aspect related to facilitating learning surrounded creating a positive environment for children on the autism spectrum. The dog mentors (dog and handler teams) achieved this through positive projection of behaviour and communication, building long-term rapport and promoting a relaxed classroom environment. Creating this positive environment was achieved by the dog mentors in the majority of schools (84%), with one school sharing, “by showing that the dog mentor is willing to enter unfamiliar environments, students model that… this can then be transferred to their own concerns and anxieties”. There were frequent mentions of how building long-term rapport (70%) helped the children to trust their dog mentor, helping them attend school. For instance, one school shared, “We have one child with ASD that we have been working with for about one year, and they refuse to attend school. More recently, they have attended school for about 1 h two times a week, and while on the school site, they sit with [the dog mentor]. A similar frequency highlighted how the dog mentors helped promote relaxed classrooms (70%) with dog mentors “providing a steady, non-judgemental presence” in class and helped to settle their pupils with “a calming presence which brings comfort to the students working in there”. Over half the schools agreed that the dog mentors promoted a more productive environment for learning for their students (58%), through creating a comfortable environment, having a “positive impact on children involved” with improved attendance specifically noted.

#### 3.1.2. Subtheme: Supported Learning

The schools reported how the dog mentors supported learning through improving engagement, focusing on tasks, and curriculum support. The majority of schools (75%) shared how the dog mentors improved engagement amongst children on the autism spectrum by creating a calm environment, “[allowing] them to engage in their learning environment more fully”, with schools reporting “significant positive outcomes of engagement in activities”. Likewise, just over half the schools (53%) reported improvement when focusing on the tasks when the dog mentor was in the classrooms with the children. They reported that the dog mentors “offered some one-on-one time as a reward” for completing tasks, which has proven useful “when no other incentives work”. Just under half of the schools (44%) were additionally able to involve the dog mentors in curriculum support, with one school making treats for their dog mentor, with ingredients “[needing] to be measured and weighed using mathematical skills in the process. This was enjoyed by the child immensely”, and another school encouraging children “to read to the dog, to help gain confidence”, with the dog being a non-judgemental listener, which likely reduced children’s anxiety about trying to read.

### 3.2. Theme 2: Mental Health

#### 3.2.1. Subtheme: Emotional Management

The most commonly reported aspect regarding mental health was how the dog mentors helped children regulate their emotions, with the majority of schools (88%) teaching ‘self-regulation through co-regulation”, which included the dog (the respondents did not mention the impact of the handler, although this may, of course, impact the observed outcomes). This improved regulation was perceived as preventing emotional outbursts, “where often [they] would have exploded,” as a result, helping the student to calm down quicker when working with the dog mentor. For instance, students are able and willing to take movement breaks or “time out of class to spend time with the dog mentor” to self-regulate. In addition to the child controlling their emotions and behaviour, during the encounter with the dog, a learning opportunity to build awareness and teach the child about the dog’s needs, “aware they have to be calm around the [dog mentor]”, also teaches the child perspective taking and further embeds the importance of their ability to regulate. By enabling children to self-regulate, they are spending more time in the classroom and on the curriculum-related tasks set. Another perceived benefit by school staff resulting from the time spent with the dogs and the improvement in self-regulation is the children’s improvement in understanding constructive criticisms (30%) (as they are more able and willing to engage in discussion), and half the schools also reported an increase in resilience (53%) with children “having much calmer days knowing [the Dog Mentor] is in school”.

In addition to the improved self-regulation and related benefits, nearly all of the schools (98%) reported how the dog mentors helped reduce stress and anxiety amongst the students, evident in different transition-related situations that occur within the educational setting. For instance, the dog mentor “accompanies a student to a new classroom or unfamiliar setting”. This is one of the anecdotally discussed benefits of the dog-handler teams who are part of The Dog Mentor programme. As a result, the organisation has guidelines on how to conduct meet and greet sessions at the school gates and transitioning between classrooms safely, ensuring the welfare of the dog (no crowding/scaring the dog). In addition to stress and anxiety reduction during periods of transition, the dogs have had a similar impact on students during daily lessons, as the presence of the dogs has acted as a positive addition to the classroom, resulting in “fewer signs of stress and anxiety”. Furthermore, during exams, when students are particularly anxious and stressed, having the dogs present has allowed students to “have a break [meaning they] can return positively” and complete the assessment to the best of their ability. Although this is based on staff perception of the impact of the dogs on the students on the autism spectrum, it is based on differences that are evident to the individuals who know the child well. As a result, more rigorous research, with physiological measures (of stress) need to be conducted, which will in turn indicate the overall level of impact (i.e., is it a minimal or large reduction in stress and is it statistically or clinically significant).

#### 3.2.2. Subtheme: Support with Daily Life

Schools reported how the Dog Mentors helped support the children’s essential skills beyond school, which contributes to their growth and development. For instance, the dog mentors supported with daily structure and routine (70%), such as “meeting children at the start of the day” and “movement breaks”. The dog mentor was also involved in teaching the children responsibility (60%), “making sure the dog’s needs were fulfilled,” such as food and water bowls being full, and having increased self-awareness (46%), knowing they “must be calm around the dog”. Less commonly reported was how the dog mentor was involved in the teaching of self-care and life skills (46%) as children modelled the behaviour and related to what the dog needs to do and how that relates to what they need to do. This resulted in the staff perceiving improved outcomes in pupils understanding their own behaviour better, developing and establishing acceptance (36%), making mistakes (41%), and support with sensory issues (48%). Sensory issues were observed on a needs basis, such as one child who sought the dog mentor throughout the day as they found playtime “overwhelming”, but with some children avoiding the dog mentor when the dog was wet due to the smell of the dog and the feeling of their fur.

### 3.3. Theme 3: Healthy Relationships

#### 3.3.1. Subtheme: Communication

The dog mentors encouraged children’s communication, and this was reported to have increased. The majority of schools indicated that the dog mentors helped with social interactions (86%), “encouraging turn-taking and social interactions in structured play”. The adaptability of the dog mentors to each child helped to “reduce stimming behaviours and increase communication”, helping the children to feel better understood due to the improved ability to communicate. Furthermore, the use of praise and awards while interacting with the dogs additionally supported the children in communicating with others (69%), such as one child who to asked in a full sentence to see their dog mentor, promoting their speech. The therapeutic environment that the dog mentors provided additionally enabled another school to encourage their students on the autism spectrum who also had mutism to speak, providing calmness to reduce the children’s anxiety. The dog mentors helped in breaking down communicative barriers, which helped teachers and staff better communicate with all students with additional needs. Teaching and creating positive body movements was also highlighted within the schools (44%); however, some schools (36%) raised concerns about keeping the dog mentors safe from the unpredictability of the behaviours expressed by the pupils on the autism spectrum, reinforcing being gentle and not grabbing the dog mentor or screaming. Though the dogs were tolerating the noise, it is important that they are not placed in situations where they have to tolerate unpleasant noise and behaviour. This is in line with The Dog Mentor training, which attempts to minimise these situations to ensure the dog’s well-being.

#### 3.3.2. Subtheme: Developing Empathy

One of the largest reported aspects achieved by The Dog Mentors was the ability to promote and help the teachers to teach children on the spectrum about empathy, both within school (84%) and more generally (81%). For instance, one school noted how they involved the Dog Mentor: “talking about how [the Dog Mentor] showed us their feelings and emotions and then the similarities and differences”. Another school noted how the children “who visit their dog mentor on a regular basis developed more empathy and a calmer manner”, as well as another school noting how their students have an “increased care for others”. However, one school raised concerns about engagements where their student’s developmental level did not meet their chronological age, not engaging as well as others. It is possible that dog mentors may be best suited for children who are developing as expected for their chronological age, if the dogs are seen as a distraction by some children with greater developmental delays. On the other hand, it may be that over time, the dogs can support children with a significant delay, but different strategies need to be implemented. It is difficult to draw conclusions, and further, in-depth research looking at the intervention structure, length, and duration per session, as well as the child’s profile and areas of delay need to be explored.

## 4. Discussion

According to dog handler reports, The Dog Mentor successfully supported children with autism, using a variety of sessions, experiencing teacher-reported fewer autism-related challenges in the classroom environment. This may indicate a reduction in autism symptoms as assessed by diagnostic tools, in line with previous research (e.g., Dimolareva & Dunn, 2021), but a thorough investigation with these measures is needed to assess this with confidence. Different interventions were likely to arise due to the spectrum of characteristics autism presents, creating differing needs for each individual. This is evident within the work of the schools registered with The Dog Mentor, and this research in particular, as the schools have highlighted that they employ a different structure of sessions. This is also in line with previous research, which has compared outcomes for one-to-one therapeutic sessions and whole class engagements (CDC, 2024; Heimlich, 2001; Somervill et al., 2009). Previous concerns relating to the variability of interventions emphasised the difficulty in assessing the effectiveness and generalising the findings. However, school staff participating in this research emphasised the need for variability within AAIs to suit the different needs of children and therefore optimise any potential benefits, such as learning and emotion regulation (Limond et al., 1997; Martin & Farnum, 2002).

For instance, school staff reported that meet and greet activities encouraged children to enter schools and created positive, calm environments, promoting engagement and supporting learning. Creating this optimal environment for learning is usually expensive, but an essential step in removing a major barrier to learning for children on the autism spectrum (Naznin et al., 2023; Zents et al., 2017; Baird et al., 2023). Therapeutic interventions from The Dog Mentor, such as regulation support, improved the children’s mental health, providing structure to their daily life, encouraging resilience within school, which actively contributed to their own personal growth (see also Wintermantel et al., 2024). The Dog Mentor additionally helped support school staff in their understanding and teaching of the children, such as using praise and reward techniques, helping enhance empathy towards peers, and improving communication of needs. This further facilitates learning, providing more evidence of improved social skills through better communication and empathy development within a classroom setting (Leos et al., 2022; O’Haire et al., 2014).

Unlike in previous research, where a set plan was followed by the researchers, The Dog Mentor adapted to the environment and the needs of each school and their structure, highlighting the flexibility of The Dog Mentor programme to facilitate learning in children on the autism spectrum, despite their differing experiences in learning (Benderix et al., 2006). These findings show that with The Dog Mentors’ training based on positive reward, empathy, respect for the animal, including encouraging children’s self-regulation prior to interaction, improvements in learning, mental health, and encouragement of healthier relationships were observed by teachers and Dog Mentor staff.

## 5. Limitations

Although these findings are promising and shine light on the types of outcomes that seem to be improved because of The Dog Mentor’s dogs and handlers, it is important to treat them with caution. The findings are based on the perception of handlers, and as a result, the data are subjective and qualitative. Furthermore, the initial checklist asked respondents to indicate which positive outcomes they had achieved (e.g., reduced stress, improved engagement), without offering neutral or negative outcomes, which may have further biassed the responses towards the perceived benefits. The data were collected by asking any of the enrolled schools to complete the questionnaire if they wished to do so, with no incentive, to ensure handlers were not forced to participate. Of the 600+ schools, 58 responses were collected. This may have resulted in the exclusion of some schools (e.g., fewer staff, higher level of challenges within their children), which would raise the question of whether the data is representative of all schools. Furthermore, likely, the handlers who did not see a benefit or had not included the dogs with neurodivergent students did not complete the questionnaire, as if they felt they did not have anything to share. As a result, we may see more positive findings in these outcomes. Although these findings show promise, it is important to assess whether this effectiveness is seen through more schools and assess how long it takes to see the differences in a standardised measure, with a more scientifically rigorous design. With this in mind, it is important to take these exploratory findings with caution. For the research team, these initial findings will guide our planning of a larger, rigorous research plan to evaluate The Dog Mentor’s impact across schools. Within our future research, we will select standardised questionnaires to rigorously assess the perceived improvements stated here. Future research will ensure more support for the schools to participate and a longer time frame for schools to engage in the research.

## 6. Conclusions

This qualitative research indicates that The Dog Mentor staff have found the dogs to facilitate learning when integrated within a wider education curriculum provided for children on the autism spectrum. This perceived support may remove some barriers to learning through the promotion of more adaptive behaviours. These handler-stated behaviours include improved self-regulation, wellbeing and communication, as well as reduced anxiety around transition into school. This was observed by children having increased attendance at schools through the support of the dogs during periods of transition.

Through a variety of different sessions, the dogs were perceived to have facilitated learning, potentially developing children’s emotional self-regulation and relationships with others. The findings from this study have added to knowledge about AAS/AAI for children on the autism spectrum in schools, highlighting the potential importance of variety and flexibility for dog-assisted support for meeting individual needs.

Future research should investigate the use of AAS/AAI for neurodivergent children experiencing trauma and loss, outside and within learning environments. Though not a common finding within this research, it was highlighted within responses from two schools how a dog mentor can help children with bereavement and trauma support. Not only does trauma directly impact learning, but childhood trauma is associated with an increased risk of suicide. This is a particular concern as people on the autism spectrum are at a higher risk of suicide (Steane, 2019; Yao et al., 2023; Newell, 2021). Due to the small number of occurrences in this study, future investigation into how The Dog Mentor may be suited to support children who have experienced trauma should be undertaken, potentially finding effective ways of support.

## Figures and Tables

**Table 1:** The frequency of schools (N = 58) using The Dog Mentor intervention techniques.

Intervention	Frequency	Success Rate
Therapeutic approach (regulation support, ELSA)	40	85%
One-on-one (individual support)	25	84%
Curriculum support (reading support)	23	86%
Meet and Greet (gate duty, helping students into school)	20	95%
Group/class sessions (classroom support)	16	87%
Transitions (changing classes, leaving school)	12	91%
Praise and Reward (positive reward for behaviour)	11	100%
Responsibilities (walks, filling water bowl)	10	100%
Own timetable (set schedule for dog)	8	62%
Free roam/access (free access to the dog for students)	6	66%
Playground access (dog available at breaktime)	2	100%

## Data Availability

Upon publication of the paper, anonymous data will be available on the University of Lincoln Repository.
